# Mitogen Activated Protein Kinase Activated Protein Kinase 2 Regulates Actin Polymerization and Vascular Leak in Ventilator Associated Lung Injury

**DOI:** 10.1371/journal.pone.0004600

**Published:** 2009-02-25

**Authors:** Mahendra Damarla, Emile Hasan, Adel Boueiz, Anne Le, Hyun Hae Pae, Calypso Montouchet, Todd Kolb, Tiffany Simms, Allen Myers, Usamah S. Kayyali, Matthias Gaestel, Xinqi Peng, Sekhar P. Reddy, Rachel Damico, Paul M. Hassoun

**Affiliations:** 1 Department of Medicine, Johns Hopkins University School of Medicine, Baltimore, Maryland, United States of America; 2 Department of Medicine, Tufts University School of Medicine, Boston, Massachusetts, United States of America; 3 Department of Biochemistry, Medical School of Hannover, Hannover, Germany; 4 Department of Environmental Health Sciences, Johns Hopkins Bloomberg School of Public Health, Baltimore, Maryland, United States of America; University of Giessen Lung Center, Germany

## Abstract

Mechanical ventilation, a fundamental therapy for acute lung injury, worsens pulmonary vascular permeability by exacting mechanical stress on various components of the respiratory system causing ventilator associated lung injury. We postulated that MK2 activation via p38 MAP kinase induced HSP25 phosphorylation, in response to mechanical stress, leading to actin stress fiber formation and endothelial barrier dysfunction. We sought to determine the role of p38 MAP kinase and its downstream effector MK2 on HSP25 phosphorylation and actin stress fiber formation in ventilator associated lung injury. Wild type and MK2^−/−^ mice received mechanical ventilation with high (20 ml/kg) or low (7 ml/kg) tidal volumes up to 4 hrs, after which lungs were harvested for immunohistochemistry, immunoblotting and lung permeability assays. High tidal volume mechanical ventilation resulted in significant phosphorylation of p38 MAP kinase, MK2, HSP25, actin polymerization, and an increase in pulmonary vascular permeability in wild type mice as compared to spontaneous breathing or low tidal volume mechanical ventilation. However, pretreatment of wild type mice with specific p38 MAP kinase or MK2 inhibitors abrogated HSP25 phosphorylation and actin polymerization, and protected against increased lung permeability. Finally, MK2^−/−^ mice were unable to phosphorylate HSP25 or increase actin polymerization from baseline, and were resistant to increases in lung permeability in response to HV_T_ MV. Our results suggest that p38 MAP kinase and its downstream effector MK2 mediate lung permeability in ventilator associated lung injury by regulating HSP25 phosphorylation and actin cytoskeletal remodeling.

## Introduction

Acute lung injury (ALI) is a devastating illness with an annual incidence of 200,000 in the United States and a mortality rate of 40% [1]. Most commonly seen in the setting of sepsis, ALI is a complex syndrome marked by increased vascular permeability resulting in tissue edema and profound hypoxia [2]. Mechanical ventilation (MV), a mainstay treatment for ALI, potentially contributes to and worsens permeability by exacting mechanical stress on various components of the respiratory system causing ventilator-associated lung injury (VALI) [3], [4]. A recent trial demonstrated a significant improvement in survival in patients ventilated with low (LV_T_) compared to high tidal volumes (HV_T_) [5]. Other than ventilating at lower tidal volumes, which presumably imparts lower mechanical stress, there is little mechanistic understanding of the pathophysiology and no directed therapies for VALI.

Mitogen activated protein (MAP) kinases are a family of stress activated enzymes (p38 MAP kinase, JNK, and ERK1/2) that initiate signaling cascades in response to external stimuli. Several recent publications have implicated p38 MAP kinase in the pathogenesis of VALI [6], [7], [8]. In addition, our laboratory has previously shown that MAP kinase activated protein kinase 2 (MK2, immediately downstream of p38 MAP kinase) leads, when activated, to heat shock protein 27 (HSP27) phosphorylation and subsequent reorganization of the actin cytoskeleton to form stress fibers [9]. HSP27 normally prevents actin polymerization by binding to G-actin monomers. However, when phosphorylated, HSP27 loses its monomeric actin binding function leading to polymerized F-actin and stress fiber formation [10]. It is well recognized that actin cytoskeletal reorganization plays a pivotal role in mediating endothelial cell barrier function and permeability such that actin polymerization and actin stress fiber formation result in increased vascular permeability by inducing paracellular gaps [11], [12], [13], [14], [15].

*In vitro*, p38 MAP kinase mediates actin stress fiber formation, paracellular gaps [16], and endothelial barrier dysfunction [10], [14], [17], [18], via activation of its downstream effector MK2 and phosphorylation of HSP27 [9], [10]. Despite these provocative *in vitro* observations on the role of p38 MAP kinase on actin dynamics and endothelial barrier dysfunction, and *in vivo* reports associating p38 MAP kinase activation with vascular permeability in VALI [19], the contribution of downstream effectors, *i.e.* MK2 and HSP25 (the mouse homologue of HSP27), in the development of pulmonary vascular dysfunction in VALI are unknown. Therefore, we tested the hypothesis that p38 MAP kinase and its downstream effector MK2 are critical for HSP25 phosphorylation and actin stress fiber formation in VALI.

## Materials and Methods

The Johns Hopkins University Institutional Animal Care and Use Committee approved all animal protocols. Fully detailed methods and protocols are available in the online supplement, Supplemental Data S1.

### Experimental protocol and animal exposure to MV

Male C57BL/6J (wild type) mice aged 10–12 weeks (Jackson Laboratory, Bar Harbor, ME) were randomly exposed to spontaneous breathing (control), LV_T_ (7 ml/kg) or HV_T_ (20 ml/kg) MV (Harvard Apparatus, Boston, MA) up to 4 hrs with slight modifications from previously described methods [19]. For certain experiments, MK2^−/−^ mice of similar background strain were used. In general MK2^−/−^ mice are viable, fertile, grow to normal size, and do not exhibit any obvious physical or behavioral defects [20].

### Drug delivery

A subset of mice received SB203580 (p38 MAP kinase inhibitor, 2 mg/kg, IP) from Sigma (St. Louis, MO), KKKALNRQLGVAA (MK2 inhibitor, 2 mg/kg, IP) from Calbiochem (San Diego, CA) or a similar volume of vehicle (DMSO) 1 hr before exposure to MV. The dose, route and timing of these treatments were based on reported half-life for these agents, prior publications and preliminary experiments demonstrating efficacy [6], [21]. To our knowledge, the MK2 inhibitory peptide has not been used *in vivo*. As such, the dosing of this peptide was chosen based on preliminary experiments showing inhibition of HSP25 phosphorylation.

### Assessment of pulmonary capillary permeability

Evans blue dye (EBD, 20 mg/kg) dissolved in PBS containing 4% BSA was injected into the external jugular vein 60 min before termination of the experiment, and extravasated EBD concentration in lung homogenates was calculated against a standard curve and reported as µg of EBD per lung as previously described [22], [23], [24]. Wet-to-dry lung weight ratios were calculated as previously described [25].

### Immunoblot analysis

Twenty five µg of protein from lung tissue homogenates were probed for phospho-specific antibodies directed at p38 MAP kinase, MK2, and HSP25, along with anti-total antibodies as recommended by the manufacturer (p38 MAP kinase and MK2 from Cell Signaling, Boston, MA, and HSP25 from Abcam, Cambridge, MA).

### Assessment of actin polymerization

After flushing free of blood, lungs were inflated with 0.6% low-melting agarose, harvested and fixed overnight in 10% buffered formalin before being embedded in paraffin. After deparaffinization and re-hydration of fixed lung tissue sections, actin polymerization was visualized using selective fluorescent probes with very high affinity for F-actin, G-actin and nuclei (Alexa Fluor 488 conjugated phalloidin, Alexa Fluor 594 conjugated DNase I and DAPI respectively) according to manufacturer protocols, Invitrogen (Carlsbad, CA). Lung tissue sections were then visualized with confocal microscopy (Zeiss LSM 510 META, Peabody,MA), and relative intensities of F- and G-actin from low power images (20× magnification) were analyzed by the National Institutes of Health ImageJ 1.37v software.

### Statistics

Data are shown as mean±standard deviations. Since values were not normally distributed log_10_ transformations were performed to normalize the data, permitting the application of parametric statistics as described previously [26]. Comparisons between groups were performed using *t*-tests or one-way ANOVA as indicated. Intergroup differences were analyzed by Tukey's multiple comparison test. A *P* value of <0.05 was considered significant. Data were analyzed using GraphPad Prism 4. In instances when all tested conditions could not be performed on a single day, data were normalized (for each experimental condition) to control conditions performed on the same day.

## Results

### High tidal volume mechanical ventilation causes pulmonary vascular permeability

**Figure 1:**
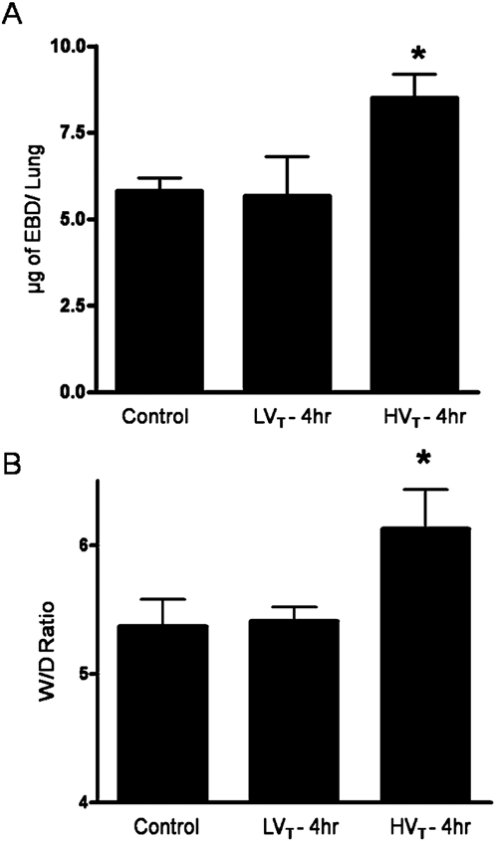
High tidal volume mechanical ventilation causes lung injury. C57BL/6J mice were randomized to spontaneous breathing (Control), LV_T_ (7 ml/kg), and HV_T_ (20 ml/kg) MV for 4 hours and EBD extravasation and wet-to-dry lung weight ratios were assessed A. The amount of EBD extravasation after MV at HV_T_ was significantly higher compared to MV at LV_T_ or spontaneous breathing (8.51 µg±0.68, 5.67 µg±1.12 and 5.82 µg±0.37 respectively), B. The wet-to-dry lung weight ratio was significantly higher after MV at HV_T_ compared to MV at LV_T_ or spontaneous breathing (6.13±0.30, 5.41±0.10 and 5.37±0.21 respectively). * *P*<0.05 (HV_T_ vs all others). N = 4–5 mice per group.

In order to determine the role of MV with varying tidal volume on lung injury, adult male wild type (WT) C57BL/6J mice were allowed to breathe spontaneously (control) or exposed to MV at 7 ml/kg (LV_T_) and 20 ml/kg (HV_T_) for 4 hours. Capillary leakage was assessed by pulmonary extravasation of EBD and wet-to-dry lung weight ratios as detailed in Materials and Methods. As shown in Figure 1, there was a significant increase in EBD extravasation and wet-to-dry lung weight ratios in animals exposed to HV_T_ as compared to LV_T_ or spontaneous breathing. Exposure of animals to LV_T_ on the other hand caused no changes in these values as compared to controls (Figure 1).

### High tidal volume mechanical ventilation activates the p38 MAP kinase-MK2-HSP25 signaling cascade

**Figure 2:**
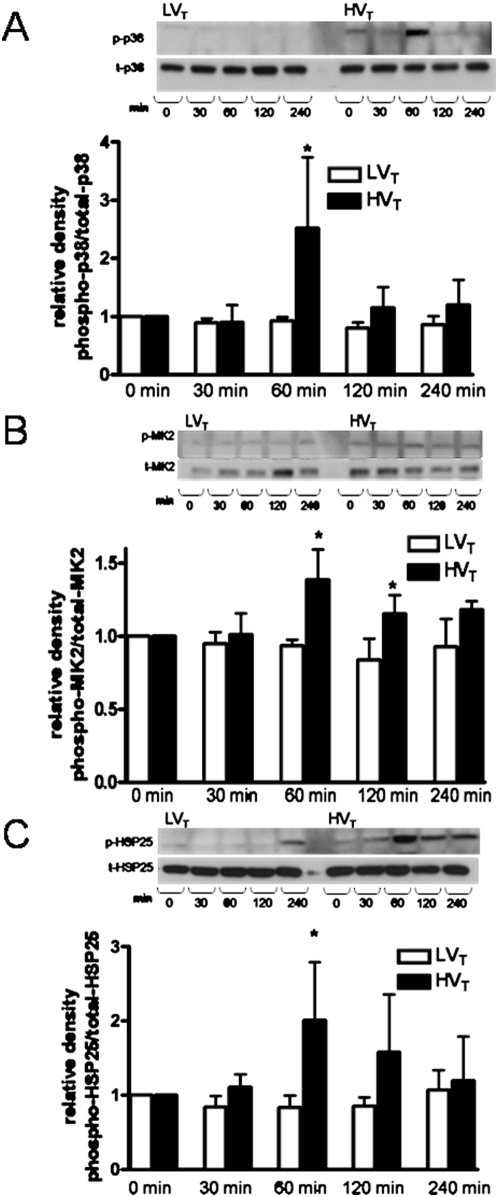
High tidal volume mechanical ventilation activates the p38 MAP kinase-MK2-HSP25 signaling cascade. C57BL/6J mice were randomized to MV at LV_T_ (7 ml/kg) and HV_T_ (20 ml/kg) for 0, 30, 60, 120 and 240 minutes after which lungs were harvested for protein analysis. Lung tissue homogenates were immunoblotted for phosphorylated and total isoforms of p38 MAP kinase, MK2 and HSP25. A. A representative Western blot indicates an increase in the phosphorylated isoform of p38 MAP kinase with MV at high tidal volumes as compared to low tidal volumes. Densitometric analysis confirms that the ratio of phospho-p38 MAP kinase/total-p38 MAP kinase peaks at 60 min of HV_T_ MV. B. A representative Western blot indicates an increase in the phosphorylated isoform of MK2 with MV at high tidal volumes as compared to low tidal volumes. Densitometric analysis confirms that the ratio of phospho-MK2/total-MK2 peaks at 60 min of HV_T_ MV. C. A representative Western blot indicates an increase in the phosphorylated isoform of HSP25 with MV at high tidal volumes as compared to low tidal volumes. Densitometric analysis confirms that the ratio of phospho-HSP25/total-HSP25 peaks after 60 min of HV_T_ MV. * *P*<0.05 (HV_T_ vs LV_T_ at same time point). N = 4–5 mice per condition.

Immunoblot analysis for phospho-specific (active) and total isoforms of p38 MAP kinase, MK2 and HSP25 was performed on lung tissue homogenates obtained from wild type mice subjected to MV with LV_T_ or HV_T_ for 0 (control), 30 min, 60 min, 120 min or 240 min. As demonstrated in Figure 2, MV at HV_T_, but not LV_T_ or spontaneous breathing conditions, induces phosphorylation of p38 MAP kinase, MK2 and HSP25. Densitometric analysis reveals an initial peak of phosphorylation identified at 60 minutes for each effector of the p38 MAP kinase-MK2-HSP25 signaling cascade.

**Figure 3:**
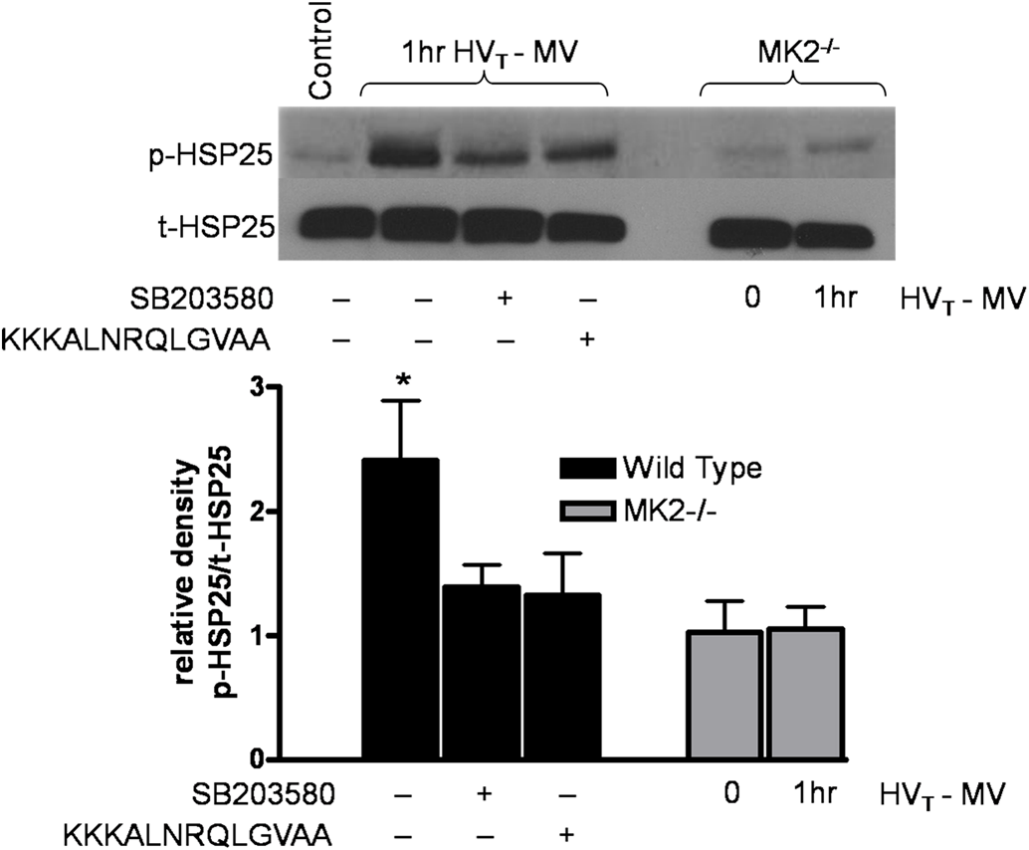
p38 MAP kinase and MK2 inhibition prevents phosphorylation of HSP25. Wild type or MK2^−/−^ mice were exposed to MV at HV_T_ for 0 or 60 minutes (peak of HSP25 phosphorylation), after which lungs were harvested for immunoblotting for phosphorylated HSP25. A subset of wild type mice were pretreated with the p38 MAP kinase inhibitor SB203580 or the MK2 inhibitor KKKALNRQLGVAA. A representative Western blot indicates that chemical inhibition of p38 MAP kinase or MK2 abrogates the phosphorylation of HSP25 and that MK2^−/−^ mice fail to phosphorylate HSP25 after exposure to HV_T_ MV. Densitometric analysis confirms that the ratio of phospho-HSP25/total-HSP25 peaks is significantly inhibited with inhibition of p38 MAP kinase and MK2 after 60 min of HV_T_ MV. * *P*<0.05 (vehicle vs wild type counter parts). N = 3–4 mice per condition.

Since our prior work had shown that HSP27 phosphorylation was necessary for stress fiber formation *in vitro* [9], we next sought to determine the role of p38 MAP kinase and MK2 on HSP25 (murine homologue of HSP27) phosphorylation on actin polymerization and stress fiber formation in response to mechanical stress *in vivo*. Mice were pretreated with the p38 MAP kinase inhibitor SB203580 (2 mg/kg, i.p.) and MK2 inhibitory peptide KKKALNRQLGVAA (2 mg/kg, i.p.) given one hour prior to MV. Immunoblot analysis for phospho- and total HSP25 was performed on lung homogenates of mice exposed to one hour of HV_T_ of MV, a time point when HSP25 phosphorylation is at its peak (Figure 2C). As shown in Figure 3, both chemical inhibitors effectively decreased HSP25 phosphorylation indicating that p38 MAP kinase and MK2 are upstream of, and required for, HSP25 phosphorylation. In parallel experiments, lung homogenates obtained from MK2^−/−^ mice exposed to MV with HV_T_ displayed normal amounts of total HSP25, however, without evidence of activation (*i.e.*, phospho-HSP25) in response to HV_T_ MV (Figure 3), further suggesting that MK2 is necessary for HSP25 phosphorylation in response to mechanical stress.

### High tidal volume mechanical ventilation induces actin polymerization

**Figure 4:**
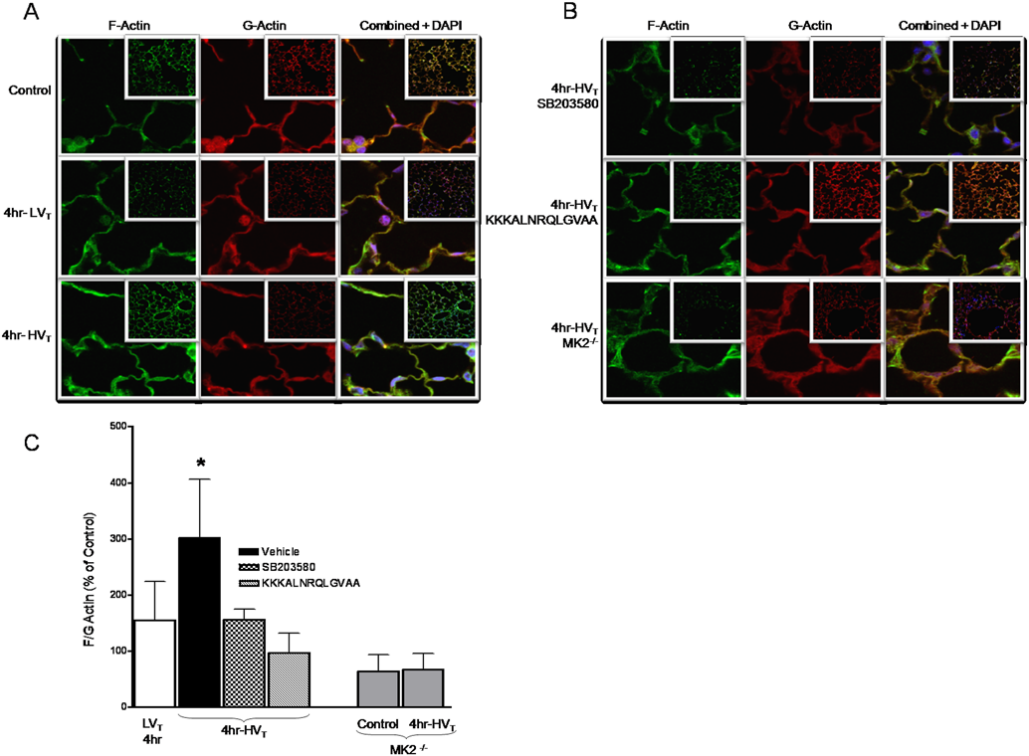
High tidal volume mechanical ventilation induces actin polymerization. Wild type or MK2^−/−^ mice were randomized to spontaneous breathing or MV at LV_T_ (7 ml/kg) and HV_T_ (20 ml/kg) for 240 minutes (4 hrs). Lung tissue sections were prepared and stained for G-actin, F-actin and nuclei as described in the Methods section. A. Representative images (100× images and inset 20×) from wild type spontaneously breathing (Control) mice or those exposed to MV at LV_T_ or HV_T_ for 4 hrs. B. Representative images (100× images and inset 20×) from wild type mice pretreated with the p38 MAP kinase inhibitor SB203580, MK2 inhibitor KKKALNRQLGVAA or MK2^−/−^ mice exposed to MV at HV_T_ for 4 hrs. Images from MK2^−/−^ mice exposed to spontaneous breathing conditions (not shown) are not different from those from mice exposed to HV_T_ MV. C. Relative intensity of F-actin and G-actin was assessed from greater than 60 random images at low power (20× magnification) per condition and graphed as F-actin to G-actin ratio to represent actin polymerization. The F-actin to G-actin ratio after MV at HV_T_ was significantly higher compared to MV at LV_T_ or pre-treatment with SB203580, KKKALNRQLGVAA (3.02±1.0, 1.55±0.69, 1.56±0.19 and 0.97±0.34, respectively). Control represents spontaneously breathing conditions. MK2-deficient mice have a lower F-actin to G-actin ratio at baseline and are unable to polymerize actin in response to MV at HV_T_ (0.63±0.29 at control conditions and 0.67±0.28 after MV at HV_T_. * *P*<0.05 (vehicle vs all others). There was no statistical difference in the F-actin to G-actin ratio between MK2^−/−^ mice under control conditions and those exposed to 4 hr of HV_T_ MV. N = 3–4 mice per group.

As our laboratory has demonstrated increased stress fiber formation with MK2 activation and HSP27 phosphorylation *in vitro* [9], we next explored the potential effect of activation of the p38 MAP kinase-MK2-HSP25 signaling pathway on actin polymerization in response to HV_T_ MV. Lung tissue sections obtained from mice exposed to MV were probed for F-actin and G-actin, as detailed in Materials and Methods. As demonstrated in Figure 4, there was significant actin polymerization, as evidenced by an increase in the F-actin to G-actin ratio, in response to MV at HV_T_ but not LV_T_. In addition, pretreatment of mice with the p38 MAP kinase inhibitor SB203580 or the MK2 inhibitory peptide KKKALNRQLGVAA significantly abrogated the increase in F-actin to G-actin ratio. Finally, MK2^−/−^ mice displayed a low F-actin to G-actin ratio at baseline (compared to wild type counterparts) and failed to increase this ratio in response to HV_T_ MV. Taken together, these results indicate that MV at HV_T_ (but not LV_T_) results in actin polymerization which is dependent on activation of p38 MAP kinase and MK2 with subsequent HSP25 phosphorylation.

### Inhibition of the p38 MAP kinase-MK2-HSP25 pathway prevents lung injury in response to high tidal volume mechanical ventilation

**Figure 5:**
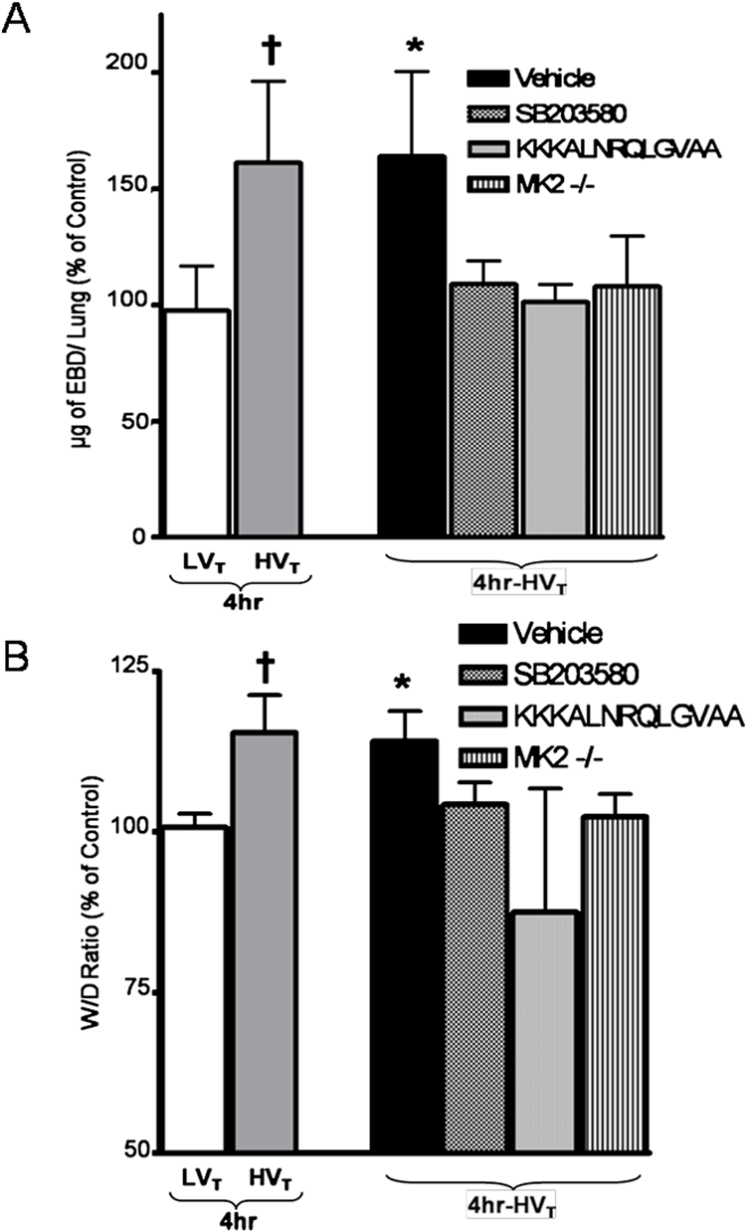
Inhibition of the p38 MAP kinase-MK2-HSP25 pathway prevents pulmonary capillary permeability in response to high tidal volume mechanical ventilation. Wild type and MK2^−/−^ mice were randomized to spontaneous breathing (Control) or HV_T_ MV for 4 hours, and EBD extravasation and wet-to-dry lung weight ratios were assessed. A subset of wild type mice were pretreated with vehicle (DMSO), the p38 MAP kinase inhibitor SB203580 or the MK2 inhibitor KKKALNRQLGVAA. A. The amount of EBD extravasation after MV at HV_T_ was significantly higher in mice treated with vehicle compared to mice treated with SB203580, KKKALNRQLGVAA or untreated MK2^−/−^ mice (1.64±0.35 Vs. 1.08±0.1, 1.01±0.07 and 1.06±0.21 respectively), EBD extravasation after MV at LV_T_ and HV_T_ for 4 hr without treatment is also shown for comparison. B. The wet-to-dry lung weight ratio after MV at HV_T_ was significantly higher in mice treated with vehicle compared to treatment with SB203580, KKKALNRQLGVAA or MK2^−/−^ untreated mice (1.15±0.04 Vs. 1.04±0.03, 0.87±0.19 and 1.02±0.03, respectively). Wet-to-dry lung weight ratio after MV at LV_T_ and HV_T_ for 4 hr without treatment is also shown for comparison. EBD extravasation and wet-to-dry lung weight ratios were similar in MK2^−/−^ mice and wild type mice under spontaneous breathing conditions (data not shown). * *P*<0.05 (Vehicle vs all others); † *P*<0.05 (HV_T_ vs LV_T_). N = 4–6 per group.

We then sought to test the role of p38 MAP kinase-MK2-HSP25 activation and resultant actin polymerization on the development of pulmonary capillary leakage. Mice pretreated with SB203580, KKKALNRQLGVAA or vehicle were exposed to 4 hr of HV_T_ MV after which capillary leakage was assessed. As shown in Figure 5, inhibition of p38 MAP kinase with SB203580 and MK2 with KKKALNRQLGVAA prevented the increase in EBD extravasation and wet-to-dry lung weight ratio as compared to vehicle treatment. In addition, MK2^−/−^ mice were resistant to pulmonary capillary leakage in response to HV_T_ MV.

## Discussion

In summary, our results demonstrate that mechanical stress imparted by HV_T_ MV causes significant pulmonary capillary permeability, as compared to LV_T_ MV and spontaneously breathing conditions, in an *in vivo* murine model of VALI. We provide evidence that MV at HV_T_, but not LV_T_ or spontaneous breathing conditions, results in activation of the p38 MAP kinase-MK2-HSP25 signaling pathway and that inhibition of p38 MAP kinase or its downstream effector, MK2, prevents pulmonary capillary permeability in response to HV_T_ MV. We further demonstrate that prevention of pulmonary vascular leakage is associated with decreased phosphorylation of HSP25 and inhibition of actin polymerization.

We have previously demonstrated using a similar murine model of VALI that high tidal volume MV without additional stimulus such as LPS (*i.e.*, one hit model) causes significant pulmonary capillary leakage in the absence of pulmonary inflammation (*e.g.*, neutrophil infiltration) [6], [19], [27]. Other groups, also using a similar model of VALI, have demonstrated that neutrophil influx occurs only after prolonged MV at HV_T_, i.e., eight hours of exposure [7]. Compared to LV_T_ and spontaneously breathing conditions, HV_T_ MV for four hours causes significant pulmonary capillary leakage as assessed by pulmonary extravasation of Evans blue dye and an increase in wet-to-dry weight lung ratio.

A potential role for p38 MAP kinase in VALI has been previously demonstrated by us and other investigators [6], [7], [8], [19]. We have recently shown that inhibition of p38 MAP kinase prevents alveolar cell apoptosis as well as activation of the enzyme xanthine oxidoreductase, a generator of oxygen radicals [6], [19]. Dolinay *et al* recently reported that deletion of MAPK kinase 3 (MKK3, an upstream activator of p38 MAP kinase), although sufficient in diminishing markers of apoptosis, was in fact not protective of lung injury (as assessed by broncho-alveolar lavage fluid protein levels, EBD extravasation and wet-to-dry lung weight ratios) upon exposure to injurious MV [7]. Since p38 MAP kinase is activated by both MKK6 and MKK3 [28], the activity of p38 MAP kinase in response to HV_T_ MV is likely incompletely suppressed, if at all, in the context of MKK3 deficiency alone, and was not evaluated in the described work [7]. Our results clearly show that inhibition of p38 MAP kinase with SB203580 is effective, as evidenced by decreased phosphorylation of downstream effectors (Figure 3), and is protective against HV_T_ MV induced increases in pulmonary capillary leakage. Furthermore, pharmacologic inhibition (*i.e.*, treatment with the peptide KKKALNRQLGVAA) or genetic disruption (*i.e.*, use of MK2^−/−^ mice) of MK2 is also protective against pulmonary capillary permeability in response to HV_T_ MV.

We have previously shown that over-expression of constitutively active MK2 in endothelial cells results in increased stress fiber formation and paracellular gaps, an effect that is blocked by overexpressing dominant negative MK2 [9]. Additionally, overexpressing a phosphomimicking mutant HSP27 (non-murine homologue of HSP25), the downstream effector of MK2, causes formation of stress fibers and paracellular gaps [9]. Visualization and quantification of actin stress fibers from *in vivo* studies has been thus far technically very challenging and, to our knowledge, has not been reported. Monomeric G-actin is known to polymerize to form filamentous F-actin [10], [29]. Since actin stress fibers are bundles of actin filaments [30], [31], we elected to quantify the amount of filamentous actin (F-actin) relative to the amount of monomeric actin (G-actin). In the present study, we demonstrate an increase in F-actin to G-actin ratio in response to HV_T_ MV, as compared to mice ventilated at LV_T_ or spontaneously breathing mice, indicating considerable cytoskeletal remodeling and an increase in the proportion of stress fibers. Although there may be changes in actin polymerization as early as 60 min in response to activation of p38 MAPK signaling, we chose to investigate the time point corresponding to increases in vascular permeability (*i.e.*, 240 min) in order to correlate the cytoskeletal changes with endothelial barrier dysfunction, a correlation well described *in vitro* [13], [16], [32], [33], [34], Furthermore, we demonstrate that inhibiting p38 MAP kinase or MK2 prevents phosphorylation of HSP25 and actin polymerization in response to MV at HV_T_ (Figure 4). Our *in vivo* findings of increased actin polymerization are consistent with validated *in vitro* models of mechanical stress [13], [35]. Chaudhuri *et al* recently demonstrated that airway smooth muscle cells exposed to cyclic strain induced HSP27 phosphorylation and resulted in actin stress fiber formation which was prevented by the p38 MAP kinase inhibitior SB203580 or by transfecting cells with nonphosphorylatable HSP27 [35]. In addition, Birukov *et al* have demonstrated that cultured endothelial cells exposed to pathologic cyclic stretch undergo increased stress fiber formation and are significantly more sensitive to thrombin-induced paracellular gap formation and barrier dysfunction as measured by transmonolayer electrical resistance [13].

**Figure 6:**
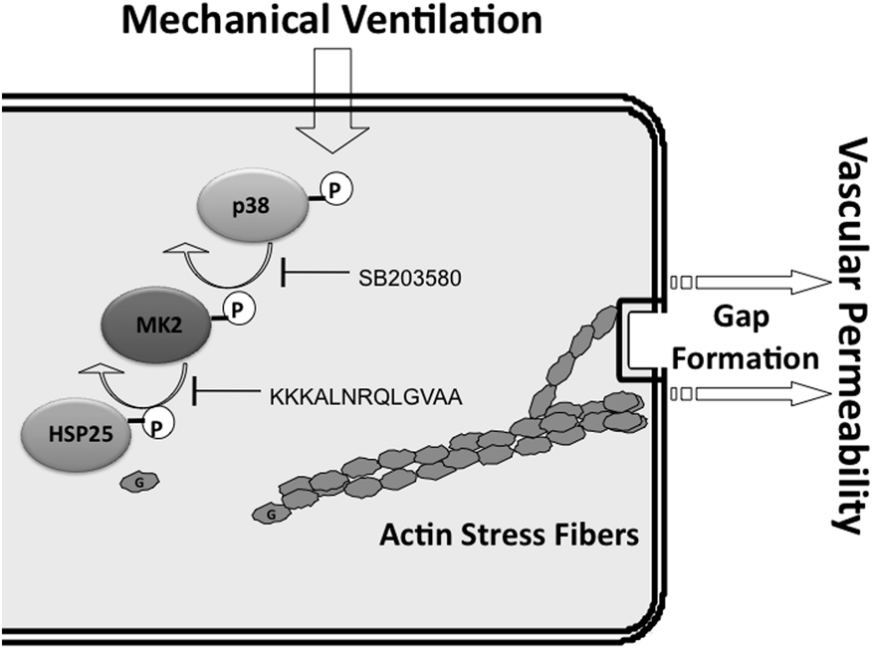
Schematic of p38 MAP kinase-MK2-HSP25 pathway. Activation of p38 MAP kinase by mechanical ventilation results in phosphorylation of MK2 and HSP25 leading to disassociation of monomeric actin from HSP25 and actin stress fiber formation mediated vascular permeability. SB203580 or KKKALNRQLGVAA treatment inhibits HSP25 phosphorylation and prevents actin stress fiber formation and vascular permeability.

Taken together with our prior work [9], our results suggest that HV_T_ MV results in phosphorylation of p38 MAP kinase, activation of MK2, and phosphorylation of HSP25, a process that causes actin to disassociate from HSP25 and polymerize to form stress fibers, which ultimately leads to paracellular gaps and increased vascular permeability. Furthermore, inhibiting p38 MAP kinase or its downstream effector MK2 prevents the phosphorylation of HSP25 and protects from vascular permeability by abrogating actin stress fiber formation and cytoskeletal rearrangement (Figure 6). It is possible that other mechanisms (*i.e.*, apoptosis, oxidant injury) are involved in endothelial barrier dysfunction in response to HV_T_ MV [36]. However, evaluating the potential role of p38 MAP kinase signaling in mediating apoptosis or oxidant injury through modulation of the cytoskeleton is beyond the scope of the present study but warrants further investigation.

Although our model creates a reproducible and reliable measure of vascular permeability as a result of mechanical stress, it lacks the ability to localize the site of vascular permeability (pre-capillary, capillary or post-capillary). Low-power field images (Figure 4) demonstrate diffuse F- and G-actin staining. However, Parker et al have shown that the microvascular segments, as opposed to the arterial or venular segments, experienced the most increase in permeability as measured by *K*_f_, the filtration coefficient, in response to high peak pressure ventilation (*i.e.*, larger tidal volumes) [37]. In addition, the alveolar-capillary unit is the major interface for MV-related mechanical stress. Finally, we have recently demonstrated in our model that alveolar epithelial and endothelial cells are the targets of mechanical stress and undergo apoptosis in response to HV_T_ MV [38]. Taken together, the capillary unit is likely the main site contributing to vascular permeability in this VALI model. Interestingly, this segmental pattern seems to be stimulus specific since post-capillary, venular, segments are mostly affected in response to ischemia-reperfusion injury [39].

It is noteworthy that the use of the p38 MAP kinase chemical inhibitor SB203580, although extensively used to address the role of p38 MAP kinase by numerous laboratories, may elicit some non-specific effects [28], [40]. However, this problem is circumvented in part by the use of fairly specific inhibitors acting at different sites (*i.e.*, SB203580 and KKKALNRQLGVAA for p38 MAP kinase and MK2, respectively) of the p38 MAP kinase-MK2-HSP25 signaling cascade. Furthermore, the use of MK2-deficient mice complements our pharmacological inhibitory studies and confirms the relevance of the p38 MAP kinase-MK2-HSP25 signaling pathway in actin polymerization and endothelial barrier dysfunction in VALI.

Although the present study strongly supports a role for p38 MAP kinase signaling via MK2 in cytoskeletal remodeling-mediated VALI, we cannot rule out the contribution of other signaling pathways (*e.g.*, myosin light chain kinase and Rho kinases) that are known to regulate cytoskeletal remodeling in vascular permeability. However, thrombin induced endothelial monolayer dysfunction (as measured by transendothelial electrical resistance) appears to be regulated by p38 MAP kinase activation in a myosin light chain phosphorylation independent manner [16]. In addition, Chaudhuri *et al* recently reported that phosphorylation of HSP27 and Rho kinase can produce stress fibers independently in response to cyclic strain [35].

In summary, this study demonstrates for the first time that inhibition of MK2 prevents lung injury in response to mechanical stress related VALI. We further provide evidence that the effects of p38 MAP kinase and MK2 on capillary permeability are partly related to cytoskeletal rearrangement and actin polymerization via HSP25 phosphorylation.

## Supporting Information

Supplemental Data S1 (0.06 MB DOC)

## References

[1] Rubenfeld GD, Caldwell E, Peabody E, Weaver J, Martin DP (2005). Incidence and Outcomes of Acute Lung Injury.. N Engl J Med.

[2] Ware LB, Matthay MA (2000). The Acute Respiratory Distress Syndrome.. N Engl J Med.

[3] Fan E, Needham DM, Stewart TE (2005). Ventilatory management of acute lung injury and acute respiratory distress syndrome.. Jama.

[4] MacIntyre NR (2005). Current Issues in Mechanical Ventilation for Respiratory Failure.. Chest.

[5] The Acute Respiratory Distress Syndrome N (2000). Ventilation with Lower Tidal Volumes as Compared with Traditional Tidal Volumes for Acute Lung Injury and the Acute Respiratory Distress Syndrome.. N Engl J Med.

[6] Le A, Damico R, Damarla M, Boueiz A, Pae HH (2008). Alveolar Cell Apoptosis is Dependent on p38-MAPK-Mediated Activation of Xanthine Oxidoreductase in Ventilator-Induced Lung Injury.. J Appl Physiol.

[7] Dolinay T, Wu W, Kaminski N, Ifedigbo E, Kaynar AM (2008). Mitogen-activated protein kinases regulate susceptibility to ventilator-induced lung injury.. PLoS ONE.

[8] Dolinay T, Szilasi M, Liu M, Choi AMK (2004). Inhaled Carbon Monoxide Confers Antiinflammatory Effects against Ventilator-induced Lung Injury.. Am J Respir Crit Care Med.

[9] Kayyali US, Pennella CM, Trujillo C, Villa O, Gaestel M (2002). Cytoskeletal Changes in Hypoxic Pulmonary Endothelial Cells Are Dependent on MAPK-activated Protein Kinase MK2.. J Biol Chem.

[10] Huot J, Houle F, Marceau F, Landry J (1997). Oxidative Stress–Induced Actin Reorganization Mediated by the p38 Mitogen-Activated Protein Kinase/Heat Shock Protein 27 Pathway in Vascular Endothelial Cells.. Circ Res.

[11] Dudek SM, Garcia JGN (2001). Cytoskeletal regulation of pulmonary vascular permeability.. J Appl Physiol.

[12] Bryan J, McVerry JGNG (2004). Endothelial cell barrier regulation by sphingosine 1-phosphate.. Journal of Cellular Biochemistry.

[13] Birukov KG, Jacobson JR, Flores AA, Ye SQ, Birukova AA (2003). Magnitude-dependent regulation of pulmonary endothelial cell barrier function by cyclic stretch.. Am J Physiol Lung Cell Mol Physiol.

[14] Bogatcheva NV, Adyshev D, Mambetsariev B, Moldobaeva N, Verin AD (2006). Involvement of microtubules, p38 and Rho kinases pathway in 2-methoxyestradiol-induced lung vascular barrier dysfunction.. Am J Physiol Lung Cell Mol Physiol.

[15] Bogatcheva NV, Verin AD (2008). The role of cytoskeleton in the regulation of vascular endothelial barrier function.. Microvasc Res.

[16] Borbiev T, Birukova A, Liu F, Nurmukhambetova S, Gerthoffer WT (2004). p38 MAP kinase-dependent regulation of endothelial cell permeability.. Am J Physiol Lung Cell Mol Physiol.

[17] Vertii A, Hakim C, Kotlyarov A, Gaestel M (2006). Analysis of Properties of Small Heat Shock Protein Hsp25 in MAPK-activated Protein Kinase 2 (MK2)-deficient Cells: MK2-DEPENDENT INSOLUBILIZATION OF Hsp25 OLIGOMERS CORRELATES WITH SUSCEPTIBILITY TO STRESS.. J Biol Chem.

[18] Galen B, Schneider HHLFC (1998). In vivo evaluation of hsp27 as an inhibitor of actin polymerization: Hsp27 limits actin stress fiber and focal adhesion formation after heat shock.. Journal of Cellular Physiology.

[19] Abdulnour RE, Peng X, Finigan JH, Han EJ, Hasan EJ (2006). Mechanical stress activates xanthine oxidoreductase through MAP kinase-dependent pathways.. Am J Physiol Lung Cell Mol Physiol.

[20] Kotlyarov A, Neininger A, Schubert C, Eckert R, Birchmeier C (1999). MAPKAP kinase 2 is essential for LPS-induced TNF-alpha biosynthesis.. Nat Cell Biol.

[21] Yan J, Hales BF (2008). p38 and JNK Mitogen-Activated Protein Kinase (MAPK) Signaling Pathways Play Distinct Roles in the Response of Organogenesis Stage Embryos to a Teratogen.. J Pharmacol Exp Ther.

[22] Moxley MA, Baird TL, Corbett JA (2000). Adoptive transfer of acute lung injury.. Am J Physiol Lung Cell Mol Physiol.

[23] Patterson CE, Rhoades RA, Garcia JG (1992). Evans blue dye as a marker of albumin clearance in cultured endothelial monolayer and isolated lung.. J Appl Physiol.

[24] Peng X, Hassoun PM, Sammani S, McVerry BJ, Burne MJ (2004). Protective effects of sphingosine 1-phosphate in murine endotoxin-induced inflammatory lung injury.. Am J Respir Crit Care Med.

[25] Itoh T, Obata H, Murakami S, Hamada K, Kangawa K (2007). Adrenomedullin ameliorates lipopolysaccharide-induced acute lung injury in rats.. Am J Physiol Lung Cell Mol Physiol.

[26] Ranieri VM, Suter PM, Tortorella C, De Tullio R, Dayer JM (1999). Effect of mechanical ventilation on inflammatory mediators in patients with acute respiratory distress syndrome: a randomized controlled trial.. Jama.

[27] Schmidt EP, Damarla M, Rentsendorj O, Servinsky LE, Zhu B (2008). Soluble guanylyl cyclase contributes to ventilator-induced lung injury in mice.. Am J Physiol Lung Cell Mol Physiol.

[28] Cuenda A, Rousseau S (2007). p38 MAP-kinases pathway regulation, function and role in human diseases.. Biochim Biophys Acta.

[29] Guay J, Lambert H, Gingras-Breton G, Lavoie J, Huot J (1997). Regulation of actin filament dynamics by p38 map kinase-mediated phosphorylation of heat shock protein 27.. J Cell Sci.

[30] Hotulainen P, Lappalainen P (2006). Stress fibers are generated by two distinct actin assembly mechanisms in motile cells.. J Cell Biol.

[31] Naumanen P, Lappalainen P, Hotulainen P (2008). Mechanisms of actin stress fibre assembly.. J Microsc.

[32] Verin AD, Birukova A, Wang P, Liu F, Becker P (2001). Microtubule disassembly increases endothelial cell barrier dysfunction: role of MLC phosphorylation.. Am J Physiol Lung Cell Mol Physiol.

[33] Garcia JGN, Liu F, Verin AD, Birukova A, Dechert MA (2001). Sphingosine 1-phosphate promotes endothelial cell barrier integrity by Edg-dependent cytoskeletal rearrangement.. J Clin Invest.

[34] Birukova AA, Birukov KG, Gorshkov B, Liu F, Garcia JGN (2005). MAP kinases in lung endothelial permeability induced by microtubule disassembly.. Am J Physiol Lung Cell Mol Physiol.

[35] Chaudhuri S, Sanyal Chaudhuri AB, Smith PG (2008). Cyclic Strain-Induced HSP27 Phosphorylation Modulates Actin Filaments In Airway Smooth Muscle Cells.. Am J Respir Cell Mol Biol.

[36] Wang Y, Huang S, Sah VP, Ross J, Brown JH (1998). Cardiac muscle cell hypertrophy and apoptosis induced by distinct members of the p38 mitogen-activated protein kinase family.. J Biol Chem.

[37] Parker JC, Yoshikawa S (2002). Vascular segmental permeabilities at high peak inflation pressure in isolated rat lungs.. Am J Physiol Lung Cell Mol Physiol.

[38] Le A, Damico R, Damarla M, Boueiz A, Pae HH (2008). Alveolar cell apoptosis is dependent on p38 MAP kinase-mediated activation of xanthine oxidoreductase in ventilator-induced lung injury.. J Appl Physiol.

[39] Grishko V, Solomon M, Wilson GL, LeDoux SP, Gillespie MN (2001). Oxygen radical-induced mitochondrial DNA damage and repair in pulmonary vascular endothelial cell phenotypes.. Am J Physiol Lung Cell Mol Physiol.

[40] Freshney NW, Rawlinson L, Guesdon F, Jones E, Cowley S (1994). Interleukin-1 activates a novel protein kinase cascade that results in the phosphorylation of hsp27.. Cell.

