# Supplementary material for: Mitogen Activated Protein Kinase Activated Protein Kinase 2 Regulates Actin Polymerization and Vascular Leak in Ventilator Associated Lung Injury
Source: PLoS One. 2009 Feb 25;4(2):e4600. doi: 10.1371/journal.pone.0004600 (PMC2643011; doi:10.1371/journal.pone.0004600)
Supplement: Supplemental Data S1 — (0.06 MB DOC) [file pone.0004600.s001.doc]

**Online Data Supplement**

**Mitogen Activated Protein Kinase Activated Protein Kinase 2 Regulates Actin Polymerization and Vascular Leak in Ventilator Associated Lung Injury**

**Mahendra Damarla, Emile Hasan, Adel Boueiz, Anne Le, Hyun Hae Pae, Calypso Montouchet, Todd Kolb, Tiffany Simms, Allen Myers, Usamah Kayyali, Matthias Gaestel, Xinqi Peng, Sekhar P. Reddy, Rachel Damico, Paul M. Hassoun.**

Materials and Methods

*Reagents.*

The p38 MAP kinase inhibitor SB203580 was obtained from Sigma (St. Louis, MO). The MK2 inhibitor KKKALNRQLGVAA was obtained from Calbiochem (San Diego, CA).

SB203580 is the prototypical inhibitor of p38 MAP kinase and inhibits the activity but not its phosphorylation (1). KKALNRQLGVAA is a 13-residue peptide that acts as a potent and selective inhibitor of MK2 (2). Phospho-specific antibodies and anti-total antibodies directed at p38 MAP kinase and MK2 were obtained from Cell Signaling (Boston, MA). Phospho-specific antibodies and anti-total antibodies directed at HSP25 were obtained from Abcam (Cambridge, MA).

*Experimental protocol and animal exposure to MV.*

Male C57BL/6J mice aged 10-12 wks (Jackson Laboratory, Bar Harbor, ME) were studied in a pathogen-free facility under a protocol approved by the Johns Hopkins Department of Laboratory Animal Medicine. Animals were first anesthetized with intraperitoneal pentobarbital (120mg/kg). A neck midline incision was then performed for exposure of the trachea to facilitate endotracheal intubation with a 20-gauge 1/2 inch long catheter (Johnson and Johnson, New Brunswick, NJ), and the animals were subjected to MV (Harvard Apparatus, Boston, MA), as previously described (3, 4) with room air for 0 (control), 0.5, 1, 2 or 4 h with LV_T_ (7 ml/kg) or HV_T_ (20 ml/kg). The respiratory rate (RR) was set at 160 breaths/min for all tidal volumes, and the dead space was adjusted to maintain arterial pH between 7.35 and 7.45. Airway pressures continuously measured during MV at 7 and 20 ml/kg revealed that end-expiratory pressures remained ~0–2 cm H_2_O throughout the 4-h period for both LV_T_ and HV_T_. A 500μl bolus of lactated ringer’s solution was given intravenously at the start of each experiment. Mean blood pressure was continuously monitored via catheterization of a femoral artery in preliminary experiments using a blood pressure monitor (Cardiomax-III) and a data acquisition system (Columbus Instruments, Columbus, OH) and remained typically ~80 mmHg. There was no difference in mean arterial pressure (MAP) between treatment groups and vehicle conditions at the beginning of mechanical ventilation. Hemodynamic values remained constant throughout the experiment and similar among all groups. More importantly, none of the mice experienced hypotension (MAP < 60mmHg) during MV. The adequacy of MV settings on gas exchange was confirmed in preliminary experiments in which arterial blood gases obtained via catheterization of a femoral artery and analyzed by an automated blood gas analyzer (Instrumentation Laboratories, Lexington, MA) revealed stable levels of arterial oxygen (PaO2 of 90-108 mmHg) and carbon dioxide (PaCO2 of 32–42 mmHg). There were no differences between treatment groups and vehicle conditions. At the end of MV, the animals were administered an intraperitoneal lethal dose of the anesthetic agent before the lungs were harvested.

For certain experiments, MK2^-/-^ mice were used. These mice, which were generated by Dr. Gaestel in similar background strain, C57BL/6, as wild type mice appear phenotypically normal, are viable, fertile, grow to normal size, and do not exhibit any obvious behavioral defects (5). Baseline respiratory compliance measurements were performed and demonstrated no significant difference between wild type and MK2^-/-^ strains, 0.042 ml/cmH_2_O and 0.044 ml/cmH_2_O, respectively. Additionally, blood gas measurements between WT mice and MK2 ^-/-^ were also similar, pH 7.38, PCO_2_ 37, PaO_2_ 107 and pH 7.40, PCO_2_ 40, PaO_2_ 106 respectively.

*Drug delivery.*

To assess the role of MAP kinase dependent pathways on MV-induced pulmonary vascular permeability, a subset of mice received SB203580 (p38 MAP kinase inhibitor, 2 mg/kg, IP), KKKALNRQLGVAA (MK2 inhibitor, 2 mg/kg, IP) or a similar volume of vehicle (DMSO) 1hr before exposure to MV. The dose, route and timing of these treatments were based on reported half-life of the agents, prior publications (6) and preliminary experiments demonstrating efficacy.

*Assessment of pulmonary capillary permeability.*

Evans blue dye (EBD, 20 mg/kg) dissolved in PBS containing 4% BSA was injected into the external jugular vein 60 min before termination of the experiment to assess vascular leak as previously described (4, 7). In brief, at the end of the experimental protocol, a thoracotomy was performed, and the lungs were perfused free of blood with PBS containing 4% BSA before being excised en bloc, blotted dry, weighed, and snap frozen in liquid nitrogen. The right lung was homogenized in PBS (1ml), incubated with 2ml of formamide (18 h, 60°C) to extract EBD, and centrifuged at 4,500 *g* for 30 min. The optical density of the supernatant was determined by spectrophotometry at 620 nm. Extravasated EBD concentration in lung homogenates was calculated against a standard curve and reported as μg EBD per lung as previously described (8).

Lung wet-to-dry weight ratio was calculated as previously described (9). In a subset of animals, wet lung weights were measured immediately after dissection, and the dried lung weight was obtained after oven drying at 60°C for a minimum of 24 hours or until the weight had stabilized.

*SDS-PAGE and immunoblot analysis.*

Aliquots from tissue homogenates were assayed for protein measurement using the Bradford protein assay and then diluted with Laemmli loading buffer for SDS-PAGE. Equal amounts of protein (25 µg) were then loaded in each well of 4–20% Trisglycine gels. After electrophoresis for 90 min at 125 V of constant voltage, the gel was blotted onto a polyvinylidene difluoride membrane by electrophoretic transfer at 25 V of constant voltage for 1 hr. The membrane was then washed, blocked with the appropriate percentage of blocking solution, and probed for phospho-specific antibodies directed at p38 MAP kinase, MK2, and HSP25, along with anti-total antibodies as recommended by the manufacturer. The immunoreactive bands were visualized using a secondary antibody conjugated to horseradish peroxidase and a chemiluminescent detection system (ECL; Amersham, Piscataway, NJ). Films were scanned using a Color Imager Scanner (Seiko Epson, Tokyo, Japan). Densitometric analysis of relative intensities of immunoreactive bands was performed by the National Institutes of Health ImageJ 1.37v software.

*Assessment of actin stress fibers.*

Actin polymerization was visualized using selective fluorescent probes with very high affinity for F- or G-actin with slight modifications of previously described methods (10). After flushing free of blood, lungs were inflated with 0.6% low-melting agarose, harvested and fixed overnight in 10% buffered formalin before being embedded in paraffin. After deparaffinization and re-hydratation, sectioned tissues were permeabilized with acetone for 10min at -20° C then incubated with Alexa Fluor 488 conjugated phalloidin (Invitrogen, Carlsbad, CA) for F-actin (diluted 1:10), Alexa Fluor 594 conjugated DNase I from Invitrogen (Invitrogen, Carlsbad, CA) for G-actin (diluted 1:500), and DAPI for nuclei (Invitrogen, Carlsbad, CA) (diluted 1:10,000). All dilutions and protocols are according to manufacturer guidelines. Sections of lung tissue were then visualized with confocal microscopy (Zeiss LSM 510 META, Peabody,MA), and relative intensities of F- and G-actin from low power images (20x magnification) were analyzed by the National Institutes of Health ImageJ 1.37v software.

*Statistics.*

Values are shown as means ± standard deviations. Since values were not normally distributed log_10_ transformations were performed to normalize the data, permitting the application of parametric statistics as described previously (11). Comparisons between groups were performed using *t*-tests or one way ANOVA as indicated. When comparing multiple groups, ANOVA was performed and if significant differences were found a Tukey's multiple comparison test was used to determine significance between individual groups. Significance was defined as *P* < 0.05. Data were analyzed using GraphPad Prism 4.

In instances when all tested conditions could not be performed on a single day, data were normalized (for each experimental condition) to control conditions performed on the same day.

REFERENCES:

1. Kumar, S., M. S. Jiang, J. L. Adams, and J. C. Lee. 1999. Pyridinylimidazole Compound SB 203580 Inhibits the Activity but Not the Activation of p38 Mitogen-Activated Protein Kinase. *Biochemical and Biophysical Research Communications* 263(3):825-831.

2. Hayess, K., and R. Benndorf. 1997. Effect of protein kinase inhibitors on activity of mammalian small heat-shock protein (HSP25) kinase. *Biochemical Pharmacology* 53(9):1239-1247.

3. Abdulnour, R. E., X. Peng, J. H. Finigan, E. J. Han, E. J. Hasan, K. G. Birukov, S. P. Reddy, J. E. Watkins, 3rd, U. S. Kayyali, J. G. Garcia, R. M. Tuder, and P. M. Hassoun. 2006. Mechanical stress activates xanthine oxidoreductase through MAP kinase-dependent pathways. *Am J Physiol Lung Cell Mol Physiol* 291(3):L345-53.

4. Peng, X., P. M. Hassoun, S. Sammani, B. J. McVerry, M. J. Burne, H. Rabb, D. Pearse, R. M. Tuder, and J. G. Garcia. 2004. Protective effects of sphingosine 1-phosphate in murine endotoxin-induced inflammatory lung injury. *Am J Respir Crit Care Med* 169(11):1245-51.

5. Kotlyarov, A., A. Neininger, C. Schubert, R. Eckert, C. Birchmeier, H. D. Volk, and M. Gaestel. 1999. MAPKAP kinase 2 is essential for LPS-induced TNF-alpha biosynthesis. *Nat Cell Biol* 1(2):94-7.

6. Yan, J., and B. F. Hales. 2008. p38 and JNK Mitogen-Activated Protein Kinase (MAPK) Signaling Pathways Play Distinct Roles in the Response of Organogenesis Stage Embryos to a Teratogen. *J Pharmacol Exp Ther*:jpet.108.139907.

7. Patterson, C. E., R. A. Rhoades, and J. G. Garcia. 1992. Evans blue dye as a marker of albumin clearance in cultured endothelial monolayer and isolated lung. *J Appl Physiol* 72(3):865-73.

8. Moxley, M. A., T. L. Baird, and J. A. Corbett. 2000. Adoptive transfer of acute lung injury. *Am J Physiol Lung Cell Mol Physiol* 279(5):L985-93.

9. Itoh, T., H. Obata, S. Murakami, K. Hamada, K. Kangawa, H. Kimura, and N. Nagaya. 2007. Adrenomedullin ameliorates lipopolysaccharide-induced acute lung injury in rats. *Am J Physiol Lung Cell Mol Physiol* 293(2):L446-52.

10. Gorska, M. M., Q. Liang, S. J. Stafford, N. Goplen, N. Dharajiya, L. Guo, S. Sur, M. Gaestel, and R. Alam. 2007. MK2 controls the level of negative feedback in the NF-kappaB pathway and is essential for vascular permeability and airway inflammation. *J Exp Med* 204(7):1637-52.

11. Ranieri, V. M., P. M. Suter, C. Tortorella, R. De Tullio, J. M. Dayer, A. Brienza, F. Bruno, and A. S. Slutsky. 1999. Effect of mechanical ventilation on inflammatory mediators in patients with acute respiratory distress syndrome: a randomized controlled trial. *Jama* 282(1):54-61.
